# Steady as they hover: kinematics of kestrel wing and tail morphing during hovering flights

**DOI:** 10.1242/jeb.247305

**Published:** 2024-08-08

**Authors:** Mario Martinez Groves-Raines, George Yi, Matthew Penn, Simon Watkins, Shane Windsor, Abdulghani Mohamed

**Affiliations:** ^1^ RMIT University, Melbourne, VIC 3000, Australia; ^2^ Department of Aerospace Engineering, University of Bristol, Bristol, BS8 1TR, UK

**Keywords:** Couplings, Agility, Locomotor dynamics, Stability, Wind hovering, Degrees of freedom

## Abstract

Wind-hovering birds exhibit remarkable steadiness in flight, achieved through the morphing of their wings and tail. We analysed the kinematics of two nankeen kestrels (*Falco cenchroides*) engaged in steady wind-hovering flights in a smooth flow wind tunnel. Motion-tracking cameras were used to capture the movements of the birds as they maintained their position. The motion of the birds' head and body, and the morphing motions of their wings and tail were tracked and analysed using correlation methods. The results revealed that wing sweep, representing the flexion/extension movement of the wing, played a significant role in wing motion. Additionally, correlations between different independent degrees of freedom (DoF), including wing and tail coupling, were observed. These kinematic couplings indicate balancing of forces and moments necessary for steady wind hovering. Variation in flight behaviour between the two birds highlighted the redundancy of DoF and the versatility of wing morphing in achieving control. This study provides insights into fixed-wing craft flight control from the avian world and may inspire novel flight control strategies for future fixed-wing aircraft.

## Teaser

**Summary:** Kestrels use a wide range of individual and coupled kinematics to maintain steady wind-hovering flights. This paper investigates the importance of these motions in this type of flight.

## INTRODUCTION

Flying birds display outstanding agility and robustness when flying in a wide variety of environmental conditions. This includes flying in turbulent and gusty conditions close to the ground (Watkins et al., 2013). Flying under these conditions can be a challenging task and birds have evolved a range of adaptations to achieve controlled flight in these environments. During non-flapping flight, birds control their flight using small adjustments of the shape and orientation of their wings and tail (Thomas and Taylor, 2001). These changes in shape are known as morphing and are believed to be an important contributor to birds' agility in flight (Lentink et al., 2007) – where agility describes ‘the ability to rapidly adjust locomotor dynamics to meet changing task demands’ (Daley, 2018). Understanding how birds use these different changes in wing and tail shape has the potential to offer inspiration for improvements to the performance and agility of similar-sized autonomous aircraft (Harvey et al., 2023).

Birds can morph their wings and tail into a very wide range of in-flight configurations. Each skeletal joint and muscle–tendon unit in the wings and tail can potentially change the appendages' shape or orientation and could be considered as a separate degree-of-freedom (DoF). However, considering each of these anatomical features separately is experimentally intractable. An alternative approach is to consider the DoF in terms of measurable shape and orientation parameters commonly used in aircraft design for which there are known flight control effects. This approach also allows comparison with avian-inspired artificial morphing wings for which detailed flight dynamic characterisation has been conducted (Di Luca et al., 2017; Ajanic et al., 2020; Chang et al., 2020; Harvey et al., 2022a,b). Using this approach, the main DoF enabling morphing in birds can be separated into two groups: those that enable wing morphing and those that allow tail morphing. Depending on the plane of morphing, the wing sub-group can be divided into flexion/extension (or sweep), pronation/supination (or twist) and elevation/depression (or dihedral) (Baliga et al., 2019). These groups can be further divided depending on the joint that is providing the motion: shoulder, elbow, wrist, etc. Those DoF actuating the tail are mainly tail yaw, tail pitch, tail roll and tail spread.

Birds use wing and tail morphing to control their orientation, which is normally defined in terms of roll, pitch and yaw. Previous research with avian-inspired morphing aircraft demonstrated that roll control can be achieved through asymmetric wing pronation/supination (Abdulrahim, 2007) and/or wing flexion/extension (Ajanic et al., 2020; Chang et al., 2020). Avian-inspired aircraft also demonstrate pitch can be controlled through modulating the pitch attitude of the tail in a similar manner to an all-moving tailplane on some aircraft (Ajanic et al., 2020) supported by the symmetric flexion/extension of the wings (Ajanic et al., 2020; Groves-Raines et al., 2022). Flight testing with radio-controlled models achieved yaw control through a combination of tail roll and pitch (Hoey, 2010), but yaw could also be controlled by the asymmetric pronation/supination of the wings (Stanford et al., 2007). Studies of bird flight kinematics suggest birds use similar mechanics (Lentink et al., 2007; Harvey et al., 2019a,b, 2022a,b; Cheney et al., 2021). However, the high anatomical DoF available to birds in comparison to the simplified DoF used in morphing aircraft suggests that bird wings are over-actuated systems (i.e. more actuator DoF than output DoF) and that birds only use a subset of the available configurations in flight. In addition, there are also very likely to be subtle morphing motions and aeroelastic shape changes involved in flight control that have yet to be characterised.

Avian flight stability can be achieved actively or passively, depending on the bird's flight behaviour. Stability is a function of aerodynamic shape, which birds can adjust in flight through morphing of their wings and tail. By changing their stability in flight, birds can potentially become more agile depending on their flight requirements. Longitudinal stability can be adjusted through the symmetric flexion/extension of the wings (Harvey et al., 2019a,b; Ajanic et al., 2020; Groves-Raines et al., 2022), as well as through the spread of their tail (Ajanic et al., 2020). The lateral stability of birds is not well characterised (Thomas and Taylor, 2001), and given that most conventional aircraft rely on large vertical stabilisers with associated drag and weight penalties, birds may offer inspiration for alternative approaches to lateral stability.

The agile yet robust flight control of birds may be enabled by the coupling of different kinematic motions. Animals are known to couple independent movements, or DoF, together to achieve specific locomotor behaviours. For example, studies of walking and running animals have revealed that there are specific kinematic couplings between DoF of the legs, body and head that are important for efficient locomotion (Anderson et al., 1995; Russell and Bels, 2001; Hall et al., 2022). In flying animals, such as birds and bats, kinematic coupling between wings, tail and body is also present in many flight behaviours (Tobalske et al., 2003; Hedrick et al., 2012; Chin and Lentink, 2019; Usherwood et al., 2020).

**Fig. 1.**
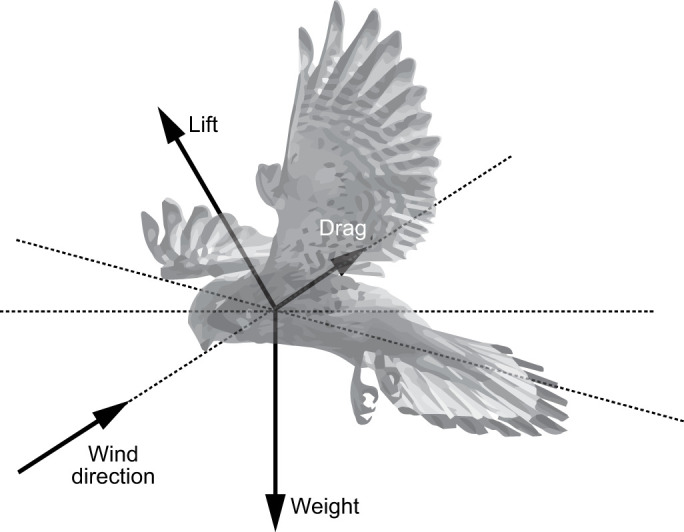
**Theory of static soaring in kestrels.** Aerodynamic forces acting on a kestrel during hovering flight in an updraft. The bird keeps its body at an angle to the wind direction, generating enough lift to balance drag and weight forces. Adapted from Videler and Groenewold (1991).

A remarkable example of the mastering of flight control and stability in birds can be observed in kestrels during their hanging flights, also known as wind hovering (Fig. 1). Kestrels, hummingbirds and some other birds can spatially hold station, keeping their body and head relatively fixed during hunting and foraging (Videler and Groenewold, 1991; Strandberg et al., 2006; Noor et al., 2014; Vejdani et al., 2019). In the presence of wind, they can exploit vertical components of the flow to achieve the necessary lift to remain ‘hanging’ in space without the need for flapping (Videler and Groenewold, 1991). Kestrels are able to perform wind hovers in relatively turbulent flows (Penn et al., 2022). Kestrel hanging flight has received little attention in the past, with some outdoor experiments which involved some flapping (Weihs et al., 1983; Videler and Groenewold, 1991), and experiments using a tilted wind tunnel (Meyers, 1993). However, the dataset presented here is the first of its kind where kestrel wind-hovering flight can be analysed through a detailed motion-tracking dataset. Understanding how kestrels manage to hold such steadiness during non-flapping flight offers the potential for useful insights for the design and control of small fixed-wing aircraft.

### Measuring avian kinematics

Avian kinematics is a challenging area to study because of the difficulty of accurately characterising the multiple DoF involved in wing and tail movements. To better understand the biomechanics of bird flight, methods are needed that can measure kinematics in an accurate and repeatable way. The recent development of camera technologies has enabled researchers to observe bird locomotion at high temporal and spatial resolution using high-speed video cameras (Crandell and Tobalske, 2015; Badger et al., 2019), track movements using motion-tracking technologies (Carruthers et al., 2007; KleinHeerenbrink et al., 2022) and even reconstruct wing surfaces using videogrammetry techniques (Durston et al., 2019; Cheney et al., 2021).

Despite these technological and experimental advances, outdoor flight experiments with birds remain a challenge because of the uncontrollability of the flight environment and the difficulty of measuring the local wind environment. Experiments with wild wind-hovering kestrels have been undertaken, but only low-resolution kinematic measurements were possible (Videler and Groenewold, 1991). Thus, most bird and bat flight kinematic studies have taken place in confined indoor spaces such as wind tunnels, where a reduced flight space can improve the repeatability of measurements (Hubel et al., 2010; Crandell and Tobalske, 2015; Badger et al., 2019; Penn et al., 2022). This approach also allows for measurements to be made from many different angles, which is especially important in the case of the complex kinematics observed in wind-hovering kestrels. However, all these studies were performed with flapping flight, which makes it difficult to decouple the small control inputs from the larger propulsion inputs.

### Aims of the study

By studying kestrels wind hovering in a relatively large wind tunnel, we aimed to reveal the main flight techniques used by these birds – whether some DoF experience higher actuation than others, whether these are coupled – and reflect on the implications for flight control and stability. Additionally, as we studied two kestrels, we aimed to explore whether there are significant differences in the way individual birds perform these wind-hovering flights.

## MATERIALS AND METHODS

### Wind-tunnel testing with live kestrels

#### Wind-tunnel set-up

**Fig. 2.**
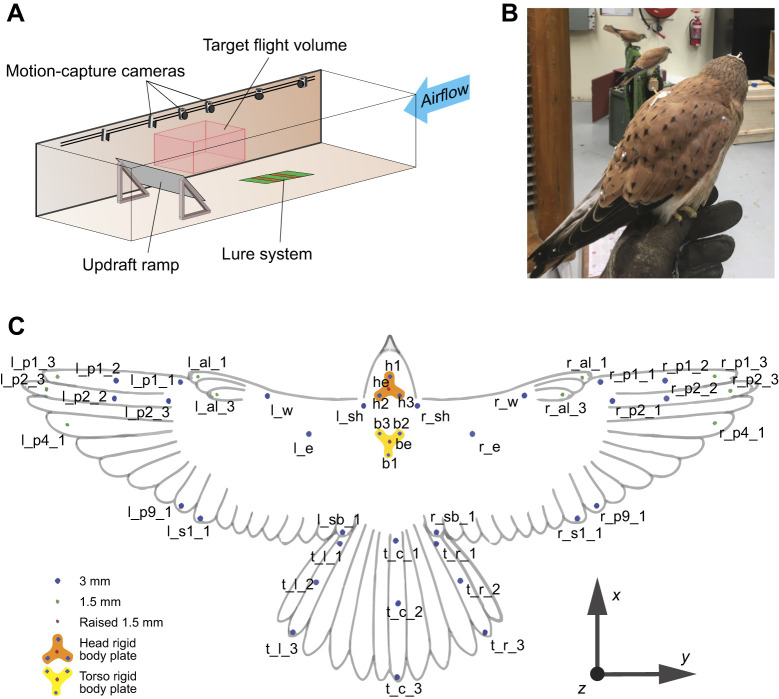
**Experimental setup.** (A) Diagram of the test section of the tunnel at the RMIT industrial wind tunnel facility, configured to facilitate bird wind-hovering flight. Modified from Penn et al. (2022). (B) A kestrel after the markers had been applied. (C) Diagram of marker distribution on the surface of the bird, showing the coordinate frame of reference for the body used to define the different degrees of freedom (DoF). Reflective markers were placed in strategic locations to reconstruct the kinematics of the birds during flight. Marker labels refer to Table S1.

The RMIT Industrial Wind Tunnel facility was adapted to facilitate the soaring and wind-hovering flight of birds (Penn et al., 2022). As shown in Fig. 2A, a ramp was placed inside the wind-tunnel section to produce a vertical flow component, which allowed kestrels to wind hover without the need for flapping. A detailed description of the wind-tunnel testing methods can be found in Penn et al. (2022). The flow in the facility was nominally smooth (turbulence intensity ∼0.8%). The wind tunnel was operated at a wind speed of 6 m s^−1^ and the flow speed over the ramp varied from 5 to 8 m s^−1^ depending on the location relative to the ramp. Two female nankeen kestrels (*Falco cenchroides* Vigors and Horsfield 1827) were trained to wind hover within a specific target volume inside the wind tunnel's test section. Several species of the genus *Falco* (kestrels) are commonly known for performing steady wind hovers even through challenging gusty environments (Videler and Groenewold, 1991).

Approval for flight testing with live birds was obtained from the RMIT Animal Ethics Committee (approval number 1804), the Victorian State Government Department of Environment, Land, Water and Planning (approval number 10008735) and the University of Bristol Animal Welfare and Ethical Review Body (UIN/20/059). Flight testing was conducted in accordance with relevant guidelines and regulations.

#### Motion tracking and marker placement

During the tests, the birds were equipped with reflective markers that were tracked using a motion-tracking camera system (Qualisys). The system was composed of 13 Qualisys Oqus 7+ infrared motion-capture cameras and 3 Miqus M5 cameras. The markers were tracked at a frequency of 300 Hz, which was more than sufficient to capture joint movement and even feather vibration. As shown in Fig. 2B,C, adhesive reflective markers were placed over the bird's feathers at strategic positions to enable the later characterisation of the kinematics in flight. A total of 50 hemispherical reflective markers were attached to each bird. Two sizes of markers were used. Markers with a diameter of 1.5 mm and a mass of ∼11 mg were applied to the tips of the primary feathers, while markers with a diameter of 3 mm and a mass of ∼15 mg were applied to the remaining locations. Their positions corresponded to anatomical joints or articulations (shoulder, elbow and wrist) or boundaries of wing surfaces (tips of wing and tail feathers). Rigid plates composed of several markers were also placed on the head and torso of the birds to gather 6 DoF data from these key body parts (Fig. 2C). The mass of these plates was 300 mg and 400 mg, respectively. Thus, the total added mass to the bird was around 1.5 g, which is less than 1% of the bird's total mass. Markers were placed by hand following the diagram shown in Fig. 2C.

#### Bird testing

The two kestrels tested were raised in captivity and were already trained to fly indoors. The birds were of very similar body mass (kestrel 1∼172 g and kestrel 2∼170 g) and size (Table S2). The training was conducted progressively over a period of approximately 3 years, for the birds to tolerate the application of markers and to perform wind hovers on demand. Food rewards were used to motivate the bird. The food was attached to a falconry lure that kept the reward hidden until the birds had performed a series of windhovers. This typically spanned a period of approximately 60 s. This system allowed the bird handler to reward the birds from behind the ramp, thus having minimal effect on the upstream flow.

#### Testing procedure

The motion-capture system was calibrated daily with a calibration wand before flight testing commenced. Calibrations with a standard deviation of less than 0.4 mm for the length of the 297.6 mm wand were used, with this error being much smaller than the size of the markers (1.5 mm diameter) and the scales of the motions recorded. Testing was conducted with one bird at a time. Two researchers applied motion-capture markers to a bird while the bird handler fed the bird with mealworms to keep it distracted. Marker placement positions were judged by eye and the same researchers applied the markers each time to keep the position as consistent as possible. However, as a result of the movement of the bird, marker placement varied between different days of testing. The process of applying markers and feeding increased each bird mass by approximately 10 g, which included the mass of the mealworms and markers. At this point the bird's mass was measured, the lure was set up, and the wind tunnel was set to the desired speed. The bird handler then entered the wind tunnel with the bird and testing commenced. Each flight typically lasted between 20 and 60 s, and consisted of a series of 10–20 wind hovers (with a duration of 0.5–2 s) separated by periods of movement from one hover location to another. The wind hover periods were the focus of this study as this allowed stabilising control motions to be studied independent of translational or flapping movements. After each flight the reward system was reset, and the process was repeated until the birds were satiated and the food rewards no longer provided sufficient motivation to continue flying. The bird's final mass was then measured, and the markers removed. Each day of testing typically yielded ∼15 flights for each bird. The mass of the birds increased approximately 10% by the end of the testing day.

### Data handling

#### Definition of a wind hover

**Fig. 3.**
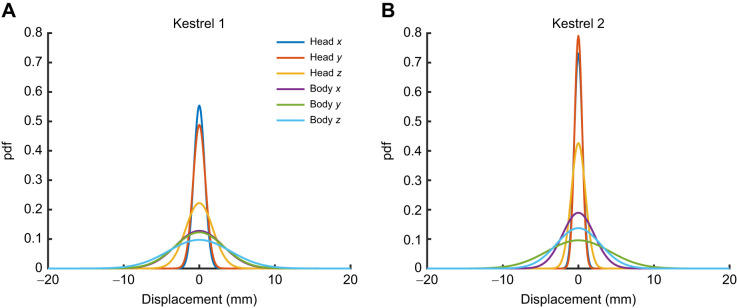
**Plots showing the displacement of head and body (torso) markers during wind hovers for the two kestrels.** (A) Kestrel 1 and (B) kestrel 2. Coordinates are given with respect to the wind-tunnel axis. The number of samples for this dataset was 174,558: 98,519 for kestrel 1 and 76,039 for kestrel 2. pdf, probability density function.

A wind hover was defined as the time frame when the birds' head markers remained within a 3-dimensional threshold of ±5 mm for a time longer than 0.5 s (Movies 2, 3). Wind hovers had a mean duration of 1.29 s, with a standard deviation of 0.65 s. For this study, only wind hovers without active flapping were selected. During wind hovers, the birds performed small wing and tail adjustments while keeping their heads relatively still. Fig. 3 illustrates the steadiness of head and torso plates for the two kestrels tested. During wind hovers, the head markers remained within the threshold of ±5 mm (95% confidence interval ±1.62 mm), whereas the body markers remained within approximately ±10 mm (95% confidence interval ±8.39 mm). Both birds exhibited similar very small head displacements during wind hovers. The actual slight movements of the head and body are believed to be smaller than those measured as a result of the additional movement of the feathers to which the rigid body plates were attached.

#### Data post-processing

Labelling of markers and tracking quality were managed using Qualisys Track Manager (QTM) software (version 2020.3). Motion-tracking data were exported from QTM as 3D position data for each marker and also as 3D position and orientation (pitch, roll and yaw angles) data for the head and torso plates. A total of 7.5% of the tracking data was not usable because of missing or shadowed markers. Tracking quality was assessed through a residual analysis, which showed markers to have normally distributed residuals with average mean of 0.49 mm and s.d. of 0.19 mm. These data were then exported to Matlab software (version R2021b) for further processing and analysis. The dataset included a total of 454 wind hovers (174,558 samples), with 265 hovers for kestrel 1 (98,519 samples) and 189 hovers for kestrel 2 (76,039 samples).

**Fig. 4.**
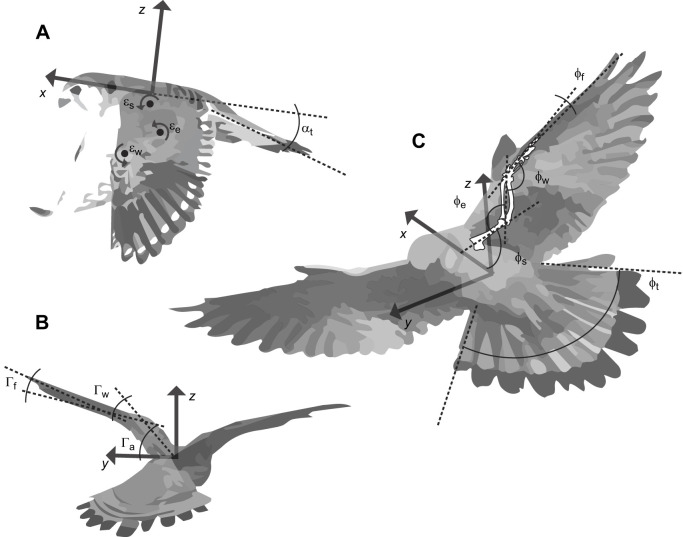
**Angle definitions for the main kinematic DoF identified in gliding kestrels.** (A) Supination and tail pitch angles, defined with respect to the body's *x*–*z* plane. (B) Wing extension and tail spread angles, defined with respect to body's *y*–*z* plane. (C) Wing elevation angles, defined with respect to the body's *x*–*y* plane. These were defined using the motion-tracking markers placed on the bird's feathers. ε_w_, wrist supination; ε_e_, elbow supination; ε_s_, shoulder supination; α_t_, tail pitch; Γ_f_, finger elevation; Γ_w_, wrist elevation; Γ_a_, arm elevation; φ_e_, elbow extension; φ_s_, shoulder extension; φ_w_, wrist extension; φ_f_, finger extension; φ_t_, tail spread. Detailed definitions of angles are given in Table S1.

By establishing angle relationships between specific markers, it was possible to define DoF that represented physical joints that the bird used to change shape or morph during flight. Fig. 4 describes the angle definitions used for this study. More detailed definitions can be found in Table S1.

Angles were defined with respect to the orientation of the rigid body plate (shown in Fig. 2C) placed on the torso of the bird. This was done so that joint angle measurements were independent of the position and orientation of the bird. Data outliers were removed and were defined as points 1.5 interquartile ranges above the upper quartile (75 percentile) and below the lower quartile (25 percentile), from Matlab's ‘rmoutliers’ function with ‘quartile’ method (https://au.mathworks.com/help/matlab/ref/rmoutliers.html), which performed best at detecting outliers after validation in QTM software. This method detected 15.5% of samples as outliers for kestrel 1 and 5.6% of samples as outliers for kestrel 2. Bootstrapping was used as a method to test the sample distribution and the effect of sample size (Table S4). It was found that even random collections of 1000 samples from the total 174,558 samples gave confidence intervals for a range of parameter means and standard deviations that were statistically indistinguishable from those obtained using the full dataset. Taking ‘wrist extension angle’ as an example, the 95% confidence intervals for the mean parameter were [106.960, 107.128] for the full dataset and [106.963, 107.127] for the bootstrap subsamples (Table S4). This indicated a more than sufficient sample size and that any differences in sample size between birds were unlikely to have any significant effect.

We characterised the kinematics used by kestrels during steady wind hovers using Matlab version R2021b software to compute metrics such as range of motion (ROM); standard deviation (s.d.), computed for each wind hover individually using Matlab's ‘std’ function; and actuation speed of the time-series dataset of angular DoF. Actuation speed outliers were removed using Matlab's ‘rmoutliers’ function – ‘Grubbs’ test, as marker shadowing could create false spikes. We then analysed the correlations and couplings between independent DoF to identify coupled kinematics used during wind hovering. All the identified kestrel DoF produced a correlation matrix using Matlab's ‘corrcoef’ function, which outputs a matrix of Pearson's correlation coefficients (*R*). The full matrix can be found in Dataset 1. The highest correlation values (over an *R*=0.6 threshold) were then further analysed to explore linearity of trends and data distribution as shown in Fig. 6. These couplings were shared between both birds, as shown in Table S3, and thus data was merged in Fig. 6. Regression lines were fitted to the data in Fig. 6 using Matlab's ‘polyfit’ function, which computes a polynomial curve of chosen order to fit a given dataset. Principal component analysis (PCA) was also used in this study to investigate couplings between DoF and gave results which closely matched the correlation analysis. Further details on the use of PCA and the results of this analysis can be found in Supplementary Materials and Methods and Fig. S1.

## RESULTS

### DoF actuation and range of motion

During wind-hovering flights, kestrels were observed to continuously perform small adjustments to the shape and orientation of their wings and tail. This can be observed in example flight videos captured during the experiments (see Movies 1, 2, 3). By comparing ROM, s.d. and actuation speeds, it was possible to identify highly actuated DoF during hovering flights.

**Fig. 5.**
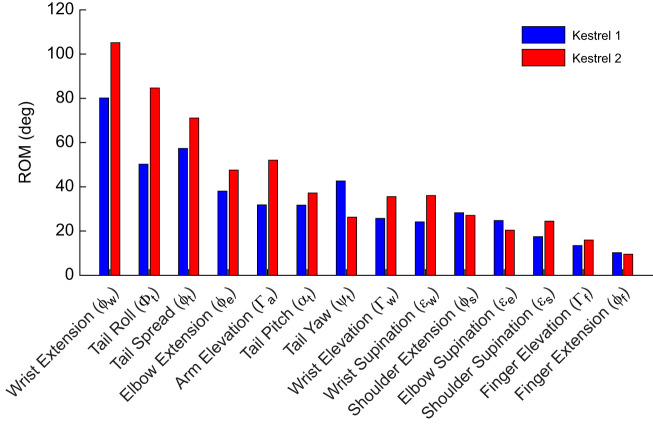
**Bar plot showing the total range of motion of the major DoF of the two kestrels during their respective wind-hovering flights.** The range of motion (ROM) was calculated between the minimum and maximum angles seen for each bird, after outliers were removed. DoF are in descending order of ROM, taking into account both birds.

ROM data were obtained through the difference between the maximum and minimum angles of each DoF. With reference to Fig. 5, wing and tail motions performed by the two birds showed similar ROM for each DoF. This similarity can also be seen in the s.d. results in Table 1. Wing DoF combined data for both left and right wings. In general, kestrel 2 showed larger ranges of motion and s.d. for all DoF than kestrel 1, which might have allowed kestrel 2 to be slightly steadier (as per Fig. 3). For both birds, wrist flexion/extension showed the highest ROM and s.d. (kestrel 1 s.d.=5.97 deg, kestrel 2 s.d.=10.01 deg). Tail roll and spread also had high ROM and s.d. – kestrel 1: s.d.=5.37 deg, kestrel 2: s.d.=8.26 deg and kestrel 1: s.d.=1.95 deg, kestrel 2: s.d.=4.54 deg, respectively.

**Table 1.**
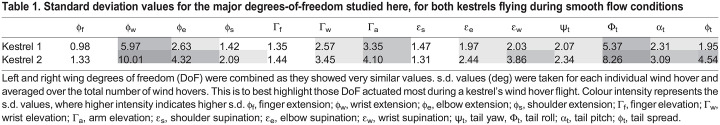
Standard deviation values for the major degrees-of-freedom studied here, for both kestrels flying during smooth flow conditions

Actuation speed data in Table 2 included wind-hovering time-series samples for both kestrels tested and were calculated based on the known sampling frequency (300 Hz) and measured changes in kinematic angle for each DoF. Actuation speeds were calculated for each wind-hovering flight individually. Table 2 shows maximum and mean actuation rates for all smooth flow flights for both tested kestrels (*n*=2), for a total of 174,558 time-series samples. A correlation can be observed between the speed of actuation and standard deviation showed in Table 1, where those DoF with highest variance are also those with the highest rates of actuation. Wrist extension of both wings, as well as tail roll actuation showed considerably higher actuation rates than the rest of the DoF. Values for maximum actuation rates presented in Table 2 were not frequent during flight tests. Mean values for actuation rates were far from their maximum, with mean values typically of 10% of their maximum rates. This indicates that the very high actuation speeds were uncommon during hovering flights. For reference, mean downstroke actuation speed calculated using cruising flapping flight kinematics from very similarly sized kestrels (Groenewegen et al., 1988) is approximately 1150 deg s^−1^, considerably higher than the mean rates measured here in wind hovering.

**Table 2.**
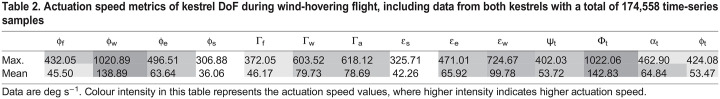
Actuation speed metrics of kestrel DoF during wind-hovering flight, including data from both kestrels with a total of 174,558 time-series samples

### Coupled kinematics during flight

Because of the high number of DoF available to these birds to change shape in flight, it is complex to analyse their individual contributions towards flight control and stability. Thus, we explored whether some of these DoF acted together and combined into repeated coupled motions.

**Fig. 6.**
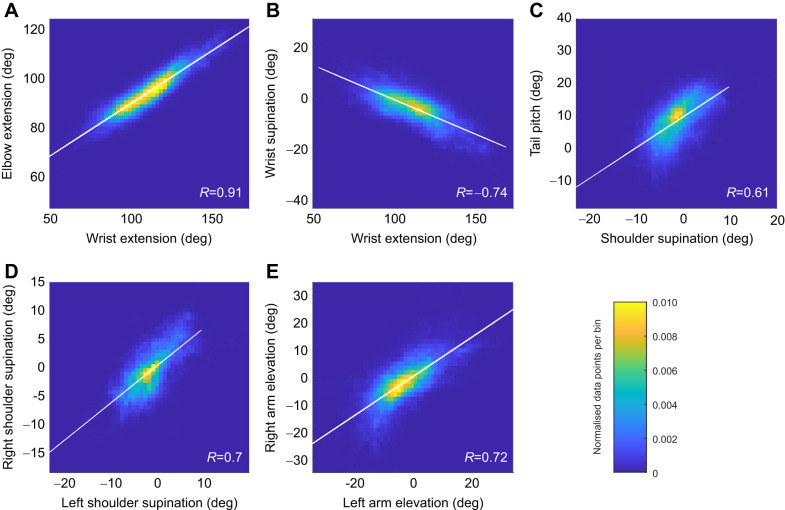
**Heatmap plots showing the distribution density of the kinematics data of the two kestrels.** Data for kestrel 1 and kestrel 2 are merged together in these plots, as couplings were shared between the two individuals. The colour bar represents the concentration of samples, normalised over the total number of data points. These plots show the most considerable kinematic couplings observed in both birds. Each plot shows the linear relationship between two independent DoF, indicating their Pearson's correlation coefficient (*R*) and a line of best fit. Couplings in A–C concentrate data for both right and left wings and thus use 349,116 data points, whereas those in D and E compare DoF between right and left wings, thus using 174,558 data points.

An exploration of the kinematic data using a cross-correlation matrix of all DoF allowed for the identification of the most dominant kinematic couplings. Fig. 6A shows that the wing elbow and wrist extensions were strongly coupled (*R*=0.91) throughout the hovering flights of both birds. These two DoF acted together during the extension/flexion motion of the whole wing. Wing wrist sweep/extension was the major DoF in terms of both ROM and s.d. This DoF was highly inversely correlated to wing wrist supination (*R*=−0.74) (Fig. 6B) for both wings and both kestrels in all wind-hovering flights analysed. This meant that as the wing was extended, the tip of the wing was twisted negatively (lower incidences). Another significant coupling was observed between the shoulder supination and the tail pitch (*R*=0.61) with respect to the bird's longitudinal body axis, as shown in Fig. 6C. This plot illustrates how the incidence of the wing correlated with the incidence of the tail.

Fig. 6 also shows further correlations that can be related to the balance of forces and moments during flight. For both birds, high correlation was seen between the twist (supination) of the right and the left wings (*R*=0.7), as seen in Fig. 6D. Similarly, the dihedral (elevation) of both wings was strongly correlated, with *R*=0.72 (Fig. 6E). Dihedral is defined here as the angle between the bird's wing and the body's lateral axis (Fig. 4B). A further coupling that was observed in the dataset was that relating to the extension of the wings and spread of the tail, which was positively correlated for both birds and had particularly high values for kestrel 1: *R*=0.70, 0.71 (Dataset 1).

### Bird behavioural differences

**Fig. 7.**
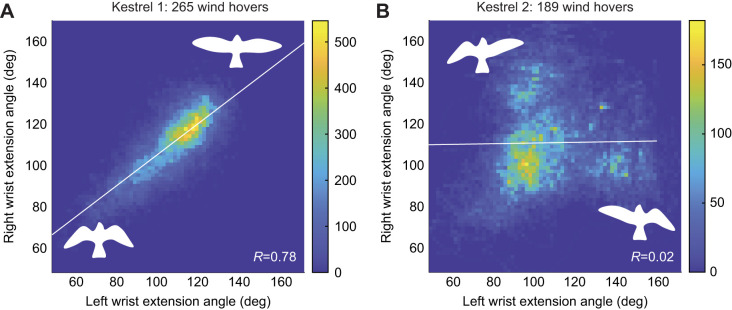
**Heatmap plots showing the correlation relationship between the wrist sweep of the left wing and that of the right wing.** (A) Kestrel 1 and (B) kestrel 2. Colour bars represent the number of samples in the pose domain. For similar numbers of wind hovers, kestrel 1 adopted much more symmetrical wing extension poses than kestrel 2.

Most correlation coefficients shared similar values between the two individuals (kestrel 1 and kestrel 2), and were analysed together in previous results. However, the correlation matrix of individual specimens showed a few differences between the behaviour of individual birds. The most significant example of this was the contrast of correlation between the left and right wrist extension, which was very high for kestrel 1 with *R*=0.78 but considerably lower for kestrel 2 with *R*=0.02. Fig. 7 shows how for very similar numbers of wind hovers, kestrel 1 flew much more symmetrically in terms of its wing extension than did kestrel 2, which adopted asymmetrical wing poses during a considerable number of flights. The poses adopted by kestrel 2 can be grouped into symmetrical and asymmetrical clusters. From the distribution of the data, it can be seen that kestrel 2 was adopting symmetrical wing sweep poses approximately half the time, and for the other half had a considerably asymmetric pose where one wing was extended and the other was tucked.

## DISCUSSION

### DoF actuation and range of motion

Previous studies on avian-inspired wing morphing may help explain the kinematics of kestrels during wind-hovering flight. Wrist flexion/extension (or sweep) was the most prominent morphing motion during flight, as indicated through metrics such as ROM (Fig. 5), s.d. (Table 1) and rates of actuation (Table 2). Flight observations showed how this DoF was actuated both symmetrically and asymmetrically with respect to both wings. For wings actuated separately (asymmetrically), this type of morphing has been previously identified as an effective technique to control roll attitude (Di Luca et al., 2017; Ajanic et al., 2020; Chang et al., 2020). Roll is also the axis most affected by atmospheric turbulence, followed by heaving motion, and both challenge controllability of small fixed-wing uncrewed air vehicles of comparable size (Watkins et al., 2010; Mohamed et al., 2014a,b). The lack of a vertical stabiliser in birds could be causing poor directional stability, which may require active control using wing sweep to avoid flight deviations in yaw. This could also explain the high actuation seen for wrist extension of both wings.

Many of the other DoF which showed high ranges of motion also appeared to be related to lateral control. Tail roll showed both a relatively large range of motion and s.d. during the wind hovering and has been associated with the ability of birds to control yaw attitude (Harvey et al., 2022a,b). The elevation/depression motion of both wings and the spread of the tail also displayed considerable actuation within wind hovers when compared with other DoF. Wing elevation/depression, also known as dihedral, is used in aircraft to tailor lateral/directional stability. In comparison to the fixed dihedral of aircraft, birds may be actively modifying this in lieu of the vertical tailplane usually used to provide directional stability in aircraft. Increasing the tail surface area through spreading the tail will enhance its effectiveness for lateral control and has also been linked with an increase in longitudinal static stability in avian-inspired aircraft (Ajanic et al., 2020). The high range of actuation of different DoF indicates that the birds are using these for flight control, but control effectiveness also needs to be considered when comparing different DoF, as the aerodynamic force contributions of some DoF may be larger than others. For example, motions resulting in changes in the incidence of the wing may have more aerodynamic effect than motions (of similar range) which change wing area. Further aerodynamic studies are needed to compare the control effectiveness of the different DoF, and are part of intended future work.

Throughout all of the flight tests the kestrels constantly performed small adjustments to the shape and orientation of their wings and tail while wind hovering. These adjustments could have been for a number of different reasons. The first is that the birds may have been statically or dynamically unstable and these constant adjustments were required to maintain their equilibrium. The second is that although the flow was nominally smooth, there was still a low level of turbulence (0.8% turbulence intensity) that may have required small motions to counteract. The third is that although the birds were in equilibrium in steady rising air, the flow distribution in the tunnel may have been such that small deviations in position moved them into slightly different airflow conditions which required them to change their pose to maintain equilibrium. None of these possibilities are mutually contradictory and all could have been occurring.

### Coupled kinematics during flight

Coupled actuation is a common feature in aircraft flight control, where different control surfaces are actuated simultaneously to achieve a control command (e.g. ailerons, elevator and rudder to achieve a banked turn).

**Fig. 8.**
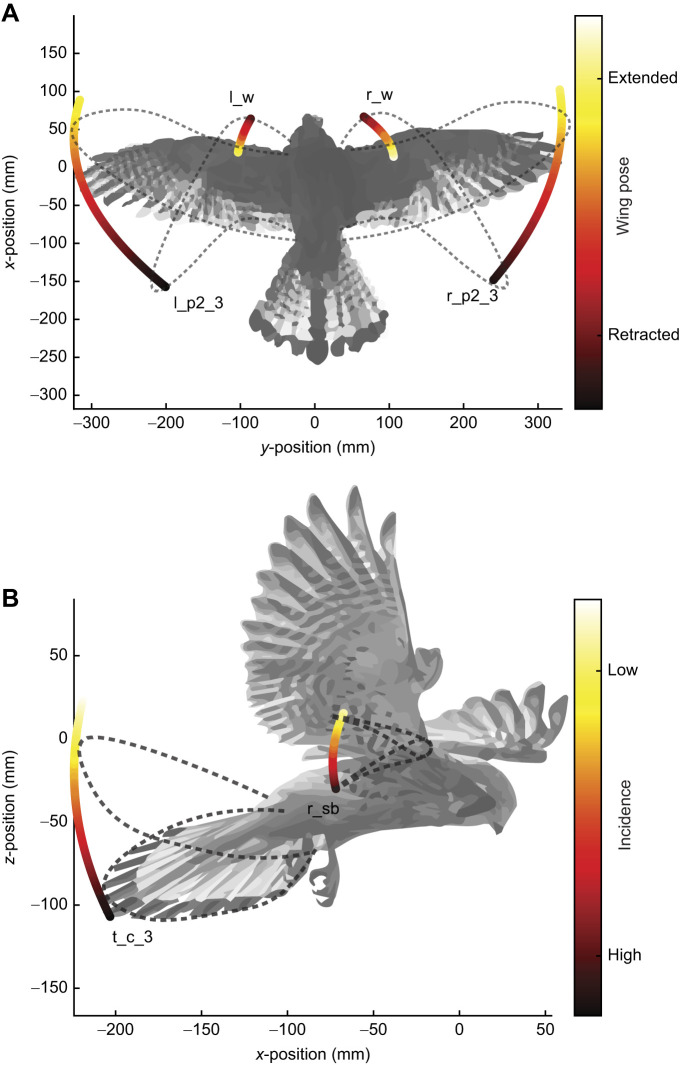
**Kinematic coupling of kestrels during flight.** (A) Diagram representing the overall motion of the wings during a flexion/extension motion of the wings, with trails representing the wrist and wing tip markers from an extended (light) and tucked (dark) wing configuration. Traces were formed by the average trajectories of markers r_w, l_w, r_p2_3 and l_p2_3 during flights. The silhouette is representative of an average pose of the kestrels in flight, with the dashed contours representing the two extreme wing extension poses. (B) Diagram representing the kinematic coupling between the incidence of wings and tail with respect to the bird's body, showing that low wing incidence correlates with low tail incidence (light) and vice versa (dark). Traces were formed by the average trajectories of markers r_sb and t_c_3 during flights. The silhouette is representative of an average pose of the kestrels in flight, with the dashed contours representing the wing chord and tail incidence extreme poses.

Wrist and elbow extension was the kinematic coupling with the highest correlation coefficient (Fig. 6A). It can be attributed to the musculoskeletal arrangement of the elbow and wrist which approximates a 6-bar-linkage mechanism (Stowers et al., 2017). The consequence of this coupling was that as the wing was retracted, the elbow moved back, and the wrist moved forwards whilst at the same time the wing tip moved backwards. As most of the mass of the wing is concentrated in the arm wing (Durston et al., 2022), this coupling is likely to keep the antero-posterior position of the centre of mass relatively constant as the wings are retracted whilst shifting the centre of area backwards. A shift in the centre of area can be used to tailor longitudinal stability in flight (Groves-Raines et al., 2022). Fig. 8A depicts a graphical representation of this kinematic coupling.

As for Fig. 6B, the negative coupling of wrist extension and wrist supination (twist) has also been found for in-flight pose data of peregrine falcons (Durston, 2019). In aircraft, a negative twist along the wing is known as ‘washout’ and is a common design choice beneficial for avoiding sudden aerodynamic stall of the outboard portion of a wing. Lowered tip incidences also reduce the magnitude of the tip vortices generated, which reduces induced drag (Green, 1995). Thus, the ‘washout’ coupling observed in kestrels for extended wing configurations may help in avoiding sudden stall and reduce induced drag. Wing retraction also reduced the overall wing surface area, which on its own would reduce the lift generated by the wing. By coupling wing retraction with an increase in tip twist, lift generated by outboard sections of the wing would have increased, which could counteract the loss of lift produced by wing retraction. Thus, we hypothesise that the coupling of wing retraction and tip twist found here may be used by kestrels to maintain constant lift generation and potentially isolate the contribution of wing extension for pitch control.

As depicted in Fig. 8B, coupling between the incidence of the wing and the incidence of the tail suggests its use in kestrels to maintain pitch equilibrium. In flight, the pitching moments generated by the wings and also by the tail must be balanced around the body's centre of gravity to achieve a trimmed or balanced state. Kestrels could use coupled wing and tail incidence to achieve a trimmed pitch attitude, but it could also aid in mitigating heave perturbations. This could be done through altering the incidence of the wing and tail relative to the wind flow, which will control the vertical lift component generated by these surfaces, allowing the birds to maintain the constant weight support necessary for a steady wind hover.

Other kinematic couplings also indicated potential equilibrium of forces required for wind hovering. Coupling of wing supination, Fig. 6D, would produce a balanced incidence of each wing with respect to the oncoming flow. Incidence relative to the flow is proportional to the amount of lift generated by each wing. Thus, having a balanced incidence would produce even lift from both wings, providing roll equilibrium. Similarly, balanced dihedral angles (elevation coupling in Fig. 6E) would avoid rolling the body relative to the wings, providing a symmetric pose which could aid flight control. A symmetric change in the dihedral of both wings could also be used as a technique to redirect the lift vector, controlling the contribution of wing lift for weight support, which would aid in trimming for different vertical wind components at different positions relative to the ramp and for maintaining a constant position in a fluctuating wind flow. Finally, both wing extension and tail spread control the surface area of the respective lifting surfaces. This type of coupled kinematics may therefore contribute to the equilibrium of forces needed for steady wind hovering.

The variable levels of scatter from a linear relationship between DoF in Fig. 6 indicated that the birds used a range of wing/tail motion combinations during flight, but on average tended to move some DoF linearly proportionally to others. The relationship between the elbow and wrist extension (Fig. 6A) had little scatter and a high correlation (*R*=0.91). This can be attributed to the musculoskeletal arrangement of the elbow and wrist, which mechanically couples these DoF, meaning that the birds have much less flexibility to change these DoF independently. In contrast, although left and right wing twist (Fig. 6D) showed an overall linear relationship (*R*=0.70), indicating equal twist angles on both wings, the scatter of the data was higher. At times there were considerable differences between the twist of the wings (up to approximately 5 deg), showing that the birds were using asymmetric wing twist at times. From a flight control perspective, this is likely to be related to when the birds flew yawed with respect to the incoming flow.

The control of motion using over-actuated musculo-skeletal systems is a long-standing question in motor control research and in particular has received a lot of attention in the human biomechanics literature (Stetter et al., 2020). A well-established theory is that a modular control architecture is used and that particular elements are controlled together and then combined with the activation of other grouped elements to produce complex motions, an idea known as ‘synergies’ (Bruton and O'dwyer, 2018). This organisation is proposed to simplify the control of these highly over-actuated systems through dimensionality reduction, with the synergies potentially existing at a neural, muscular or kinematic level. The couplings seen in the kestrel's kinematics could potentially reflect the use of synergies to simplify the task of flight control. A planned future avenue to test this theory is to explore whether some of these couplings have flight control benefits versus individually actuated DoF.

### Bird behavioural differences

Regarding Fig. 7, it was unclear why the two birds flew differently regarding their wing extension symmetry, as the two birds flew under similar conditions, both performed successful wind hovers and both were trained in the same way by the same bird handler. Both birds also maintained similar hovering positions with respect to the ramp, flying at similar flow updraft angles between 10 and 15 deg (Penn et al., 2022). Throughout the wind hovers collected, a common occurrence was for the birds to fly at a slight yaw angle with respect to the oncoming flow. Kestrel 2 may have been using a strategy involving asymmetric wing pose in order to achieve yaw equilibrium.

It is likely that some of the behavioural couplings seen have been learnt by the birds as they developed their flying technique, as opposed to being part of their innate behaviours. The different wing extension behaviours of the two birds, with very similar morphologies, under identical conditions supports this hypothesis. In raptors, learning appears to play an important role in flight using environmental flows and to be particularly important for agile foraging techniques (Ruaux et al., 2020). That both birds were able to achieve wind-hovering flights of similar steadiness through different flight techniques also shows that there are a range of possible wing and tail configurations that can be used to achieve steady wind hovering.

For equilibrium of aerodynamic forces, flying symmetrically seems a simpler solution from a human standpoint. However, even though kestrel 2 adopted asymmetric poses in flight, the versatility to modify other DoF allowed it to achieve the equilibrium of forces necessary for steady wind hovers. Deviations from linear relationships seen in most of the highlighted kinematic couplings also indicate a redundancy in terms of the number of DoF available to achieve flight control. This versatility will transfer to other flight manoeuvres and is likely a major factor in the robust and agile flight demonstrated by birds. It must be noted, however, that the small sample size limits our capability to generalise about kestrel flight behaviours.

### Conclusions

We undertook a unique experiment which enabled us to isolate avian control kinematics whereby two kestrels were trained to wind hover in a controlled wind-tunnel environment in nominally smooth flow. Small markers on the bird's wing, tail, body and head parts enabled accurate tracking of the kinematics. We aimed to identify differences between the numerous DoF involved in flight, explore whether these were combined into multi-DoF motions, and compare the flight styles of individual birds.

Wing extension featured the largest actuation of all DoF, suggesting this is a significant contributor to maintaining position during wind hovering. Most DoF that showed high actuation have been previously associated with lateral/directional stability. As birds are thought to have poor lateral/directional stability, active control using their control surfaces likely provides them with the stability necessary for wind hovering.

Coupled kinematics were identified between several DoF. The extension/flexion motions of the kestrel's wings were shown to be a combination of wrist and elbow sweep, which act together to morph the wing surface for symmetrical (longitudinal) control. During sweep, the results showed how the tips of the wings twisted positively during retracted configurations. Symmetrical movements between the left and right wings and between the wings and tail were seen throughout the flights, indicating the balancing of longitudinal aerodynamic forces during hovering manoeuvres.

Furthermore, differences in combinations of wing and tail motions were observed between the flight of the two individual birds despite holding similar levels of steadiness. This, together with the observed deviations from linearity of some of the kinematic couplings, highlights the versatility of these birds to morph their wing surfaces differently to achieve the same flight goal: here, a steady wind hover. These results suggest that birds can rely on several morphing techniques to achieve similar levels of control in different attitudes (pitch, roll, yaw).

Further work is planned to directly study the effects of these morphing techniques, but also to test the flight of kestrels and small uncrewed aerial vehicles (UAVs) in replicated atmospheric gusts to extract any potential benefits for these aircraft.

## Supplementary Material

10.1242/jexbio.247305_sup1 Supplementary information

Dataset 1. Correlation matrices showing the Pearson Correlation coefficient values of the major DoF identified. First matrix uses data for smooth flow test conditions for the two birds tested together, whereas second and third matrices refer to kestrel 1 and kestrel 2 respectively. The largest correlation values have been discussed in detail within the main publication.
