# Supplementary material for: Steady as they hover: kinematics of kestrel wing and tail morphing during hovering flights
Source: J Exp Biol. 2024 Aug 8;227(15):jeb247305. doi: 10.1242/jeb.247305 (PMC11418201; doi:10.1242/jeb.247305)
Supplement: Supplementary information [file jexbio-227-247305-s1.pdf]

Supplementary Materials and Methods

Principal Component Analysis

The complex dataset obtained here from the kinematics of kestrels during flight has a high number of variables with a very large number of samples. Analysing such type of dataset can be challenging, particularly when focusing on multivariate interactions. Principal Component Analysis (PCA) is a method commonly used for such datasets that can reduce dimensionality and increase interpretability, with minimum information loss (Jolliffe and Cadima, 2016). PCA has been used as a technique to highlight the main DoFs of biological mechanisms such as bird wings (Stowers, Matloff and Lentink, 2017) and bat wings (Riskin *et al.*, 2008). PCA breaks down a given dataset, in this case the kinematic angles formed through relationships between markers, into linear principal components. These PCs correlate to physical variables (DoFs) in different degrees. By analysing the contribution of different PCs and DoFs it is possible to simplify the kinematics of kestrels during steady wind hovers.

PCA was performed using the kinematics dataset with Matlab's own 'pca' function. The PCA analysis highlighted the dominance of wrist extension for both birds (Fig. S1), as well as tail roll and tail spread which are highlighted in Fig. 6. PCA can also show how variables are coupled or correlated together. The results showed how wing wrist and elbow extension were strongly linked, and how wrist extension and wrist supination were inversely correlated to each other. These findings support those obtained through different methods in Figure 7. Further, PCA showed behavioural differences in wrist extension between individual birds, with kestrel 1 showing very symmetrical extension/sweep poses during flight, as opposed to kestrel 2 (Fig. S2).

Journal of Experimental Biology • Supplementary information

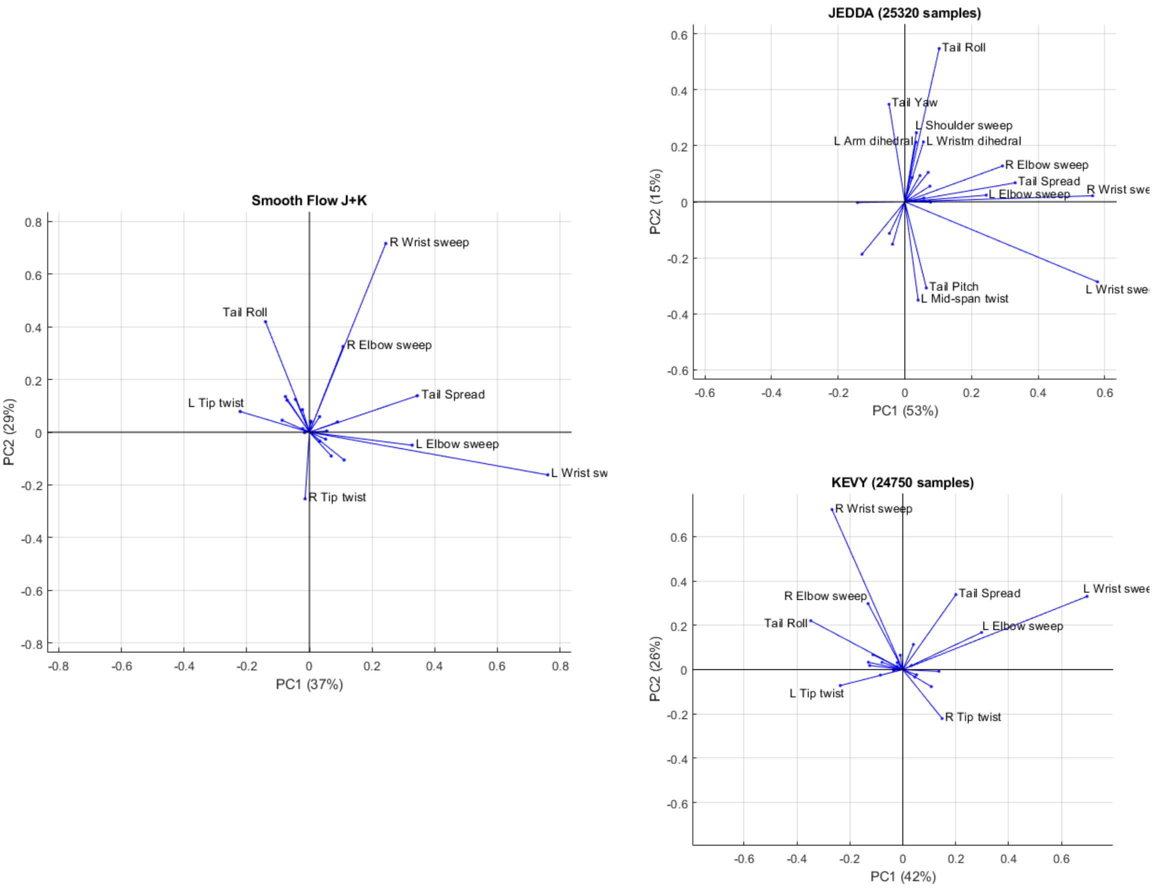

Fig. S1. A) Graphical representation of the Principal Component Analysis performed on the data for smooth flow conditions of both birds together. The overall kinematics (or overall change in morphing shape) is dominated by wing sweep/wing extension, tail spread and tail roll to some degree. A total of 66% of the overall change in shape of the bird during flight can be represented by the first two Principal Components. B) and C): Behavioural differences between B) kestrel 1 (Jedda) and C) kestrel 2 (Kevy) were also reflected on the PCA analysis. Kestrel 1 showed more symmetrical poses and hence the sweep or extension of the two wings form part of the same PC. Kestrel 2 used more asymmetrical poses where the extension of the left and right wings were not correlated.

Table S1. Kinematic angle definitions of the main DoFs actuated by the kestrels during wind hovering flights. Angles are defined through relationships between motion tracking markers placed on strategic points across the bird's body. These angles allow for the characterisation of kestrel flight kinematics.

Joint angle/DOF	Calculation
L Finger Extension	Angle l_p1_3-l_p1_1-l_w relative to body XY-plane
L Wrist Extension	Angle l_p1_1-l_w-l_e relative to body XY-plane
L Elbow Extension	Angle l_w-l_e-l_sh relative to body XY-plane
L Shoulder Extension	Angle l_e-l_sh to body X-axis relative to body XY-plane
L Finger Elevation	Angle l_p1_3-l_p1_1-l_w relative to body YZ-plane
L Wrist Elevation	Angle l_p1_1-l_w-l_e relative to body YZ-plane
L Arm Elevation	Angle l_w-l_sh to body Y-axis relative to body YZ-plane
L Shoulder Supination	Angle l_sh-l_sb_1 to body X-axis relative to body XZ-plane
L Elbow Supination	Angle l_w-l_s1_1 to l_sh-l_sb_1
L Wrist Supination	Angle l_w-l_s1_1 to l_p1_2-l_p4_1
R Finger Extension	Angle r_p1_3-r_p1_1-r_w relative to body XY-plane
R Wrist Extension	Angle r_p1_1-r_w-r_e relative to body XY-plane
R Elbow Extension	Angle r_w-r_e-r_sh relative to body XY-plane
R Shoulder Extension	Angle r_e-r_sh to body X-axis relative to body XY-plane
R Finger Elevation	Angle r_p1_3-r_p1_1-r_w relative to body YZ-plane
R Wrist Elevation	Angle r_p1_1-r_w-r_e relative to body YZ-plane
R Arm Elevation	Angle r_w-r_sh to body Y-axis relative to body YZ-plane
R Shoulder Supination	Angle r_sh-r_sb_1 to body X-axis relative to body XZ-plane
R Elbow Supination	Angle r_w-r_s1_1 to r_sh-r_sb_1
R Wrist Supination	Angle r_w-r_s1_1 to r_p1_2-r_p4_1
Tail Yaw	Angle t_c_1-t_c_3 to body X-axis relative to body XY-plane
Tail Pitch	Angle t_c_1-t_c_3 to body X-axis relative to body XZ-plane
Tail Roll	Angle t_l_3-t_r_3 to body Y-axis relative to body YZ-plane
Tail Spread	Angle t_l_1-t_l_3 to t_r_1-t_r_3 relative to body XY-plane

Table S2. Main morphological parameters of the two kestrels tested in flight. Mass data is averaged over a number of flights and days, whereas dimensional data is taken from motion tracking data: wing span is the absolute distance betwwen tip markers of each wing (r_p2_3 – l_p2_3), and body legth is the absolute distance between tail tip marker t_c_3 and head marker he. Wing area relates to a single wing and is computed using the outer markers of the wing. MAC relates to mean aerodynamic chord, and is calculated as wing area over wing span.

	K1	K2
Mass (g)	172	170
Max. Wing span (mm)	697	670
Body length (mm)	331	327
Max. Wing area (mm^2)	2.78E+04	2.97E+04
MAC (mm)	40	44

Journal of Experimental Biology • Supplementary information

Table S3. Pearson's correlation coefficients of major correlations shown in Fig. 7, for each flight test and each wing where applicable. Correlation coefficients show similar values for different tests, indicating that these couplings were shared among both birds and through a range of flight tests. These results are merged together for visualisation in Fig. 7. NaN values for flight test K1_1.1 are a consequence of multiple missing markers for this test, where several important wing markers were lost at the start of the test.

		Flight test							
		K1_1.1	K1_1.2	K1_1.3	K1_2.1	K1_2.2	K2_1.1	K2_1.2	K2_2
No. of samples		24995	26293	22330	5429	19472	19068	32693	24278
Wirst extension vs Elbow extension	L	0.93	0.94	0.93	0.89	0.87	0.98	0.96	0.91
	R	NaN	0.93	0.90	0.90	0.73	0.72	0.97	0.96
Wrist extension vs wrist supination	L	NaN	-0.79	-0.79	-0.19	-0.59	-0.93	-0.82	-0.67
	R	NaN	-0.82	-0.69	-0.29	-0.34	-0.84	-0.85	-0.73
Tail pitch vs Shoulder supination	L	NaN	0.54	0.51	0.36	0.83	0.04	0.81	0.61
	R	NaN	0.48	0.54	-0.03	0.88	0.27	0.74	0.56
R vs L shoulder supination	NA	NaN	0.84	0.55	0.28	0.83	0.42	0.80	0.68
R vs L arm elevation	NA	0.76	0.87	0.64	0.80	0.54	0.70	0.76	0.69

Table S4. Bootstrap confidence interval test performed on the kestrel kinematics dataset, for three right wing DoFs and a sample size of 174558. Test was performed for 1000, 5000 and 10000 bootstrap samples, with differences being minimal. Calculated mean and std values of the kinematics dataset fall within the confidence intervals (CI) generated through bootstrapping, indicating that this dataset is a good representation wider population.

Bootstrap Samples	1000					
	Bootstrap mean CI		True mean	Bootstrap std CI		True std
**Wrist extension**	106.963	107.127	107.044	17.771	17.895	17.834
**Elbow extension**	96.953	97.030	96.990	7.894	7.954	7.922
**Shoulder extension**	69.671	69.729	69.700	5.525	5.581	5.553
Bootstrap Samples	5000					
	Bootstrap mean CI		True mean	Bootstrap std CI		True std
**Wrist extension**	106.960	107.128	107.044	17.770	17.896	17.834
**Elbow extension**	96.951	97.026	96.990	7.893	7.951	7.922
**Shoulder extension**	69.670	69.729	69.700	5.524	5.585	5.553
Bootstrap Samples	10000					
	Bootstrap mean CI		True mean	Bootstrap std CI		True std
**Wrist extension**	106.960	107.128	107.044	17.770	17.895	17.834
**Elbow extension**	96.953	97.027	96.990	7.893	7.952	7.922
**Shoulder extension**	69.672	69.730	69.700	5.523	5.585	5.553

Dataset 1. Correlation matrices showing the Pearson Correlation coefficient values of the major DoF identified. First matrix uses data for smooth flow test conditions for the two birds tested together, whereas second and third matrices refer to kestrel 1 and kestrel 2 respectively. The largest correlation values have been discussed in detail within the main publication.

Available for download at
https://journals.biologists.com/jeb/article-lookup/doi/10.1242/jeb.247305#supplementary-data

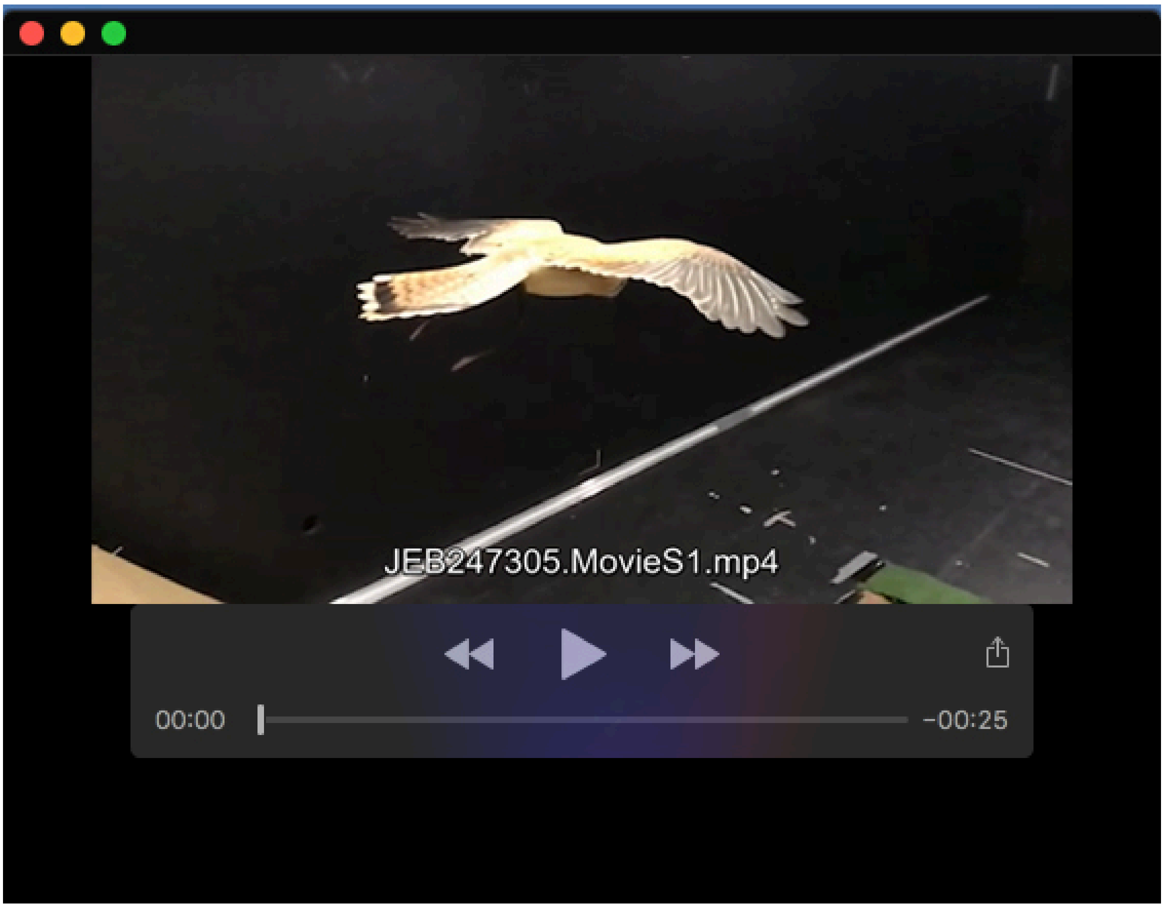

Movie 1. Slow motion video played at 12.5% of real speed, showing Kestrel 1 performing a series of wind hovering flights.

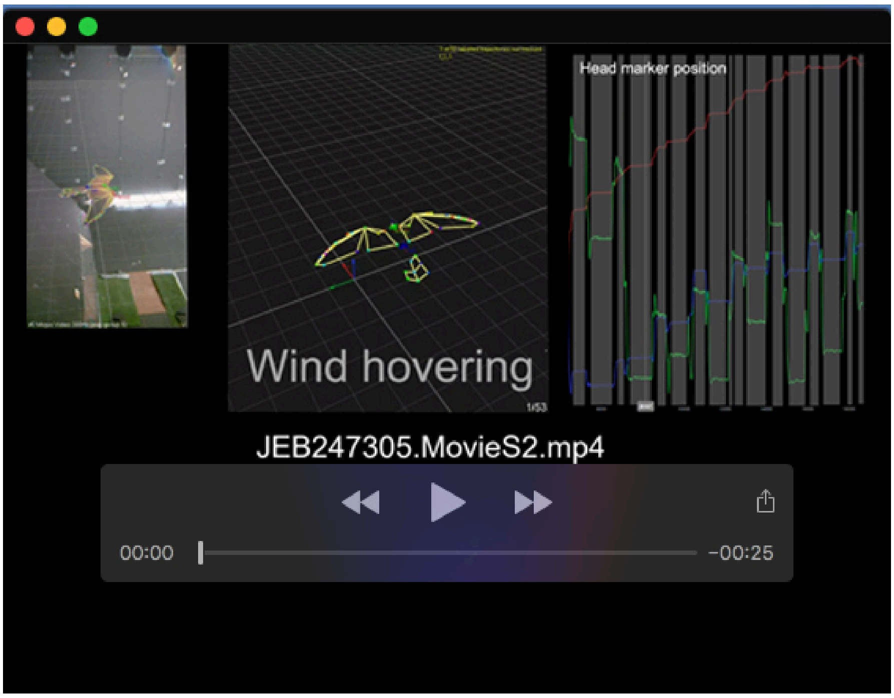

Movie 2. Real speed video of Kestrel 1 performing a flight test, showing the output geometry from the motion capture system and indicating what a wind hover is defined as.

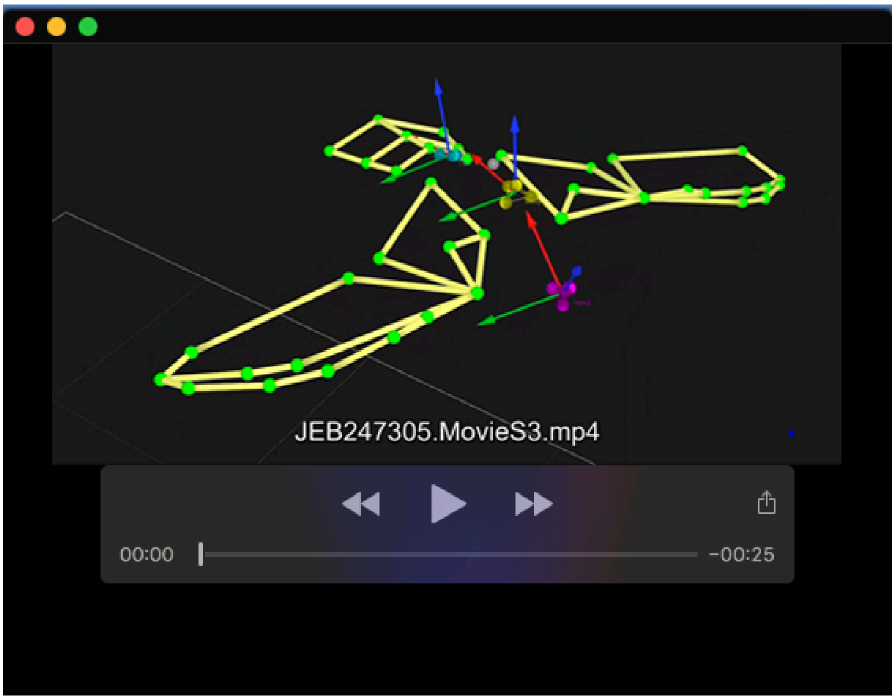

Movie 3. Slow motion video played at 10% of real speed, showing the motion capture output of Kestrel 1 during a single wind hover, demonstrating the fixation of the head markers with respect to the 3-dimensional space. Note: not all frames are showed in this video sample.

Journal of Experimental Biology • Supplementary information

References

Jolliffe, I.T. and Cadima, J. (2016) ‘Principal component analysis: a review and recent developments’, *Philosophical Transactions of the Royal Society A: Mathematical, Physical and Engineering Sciences*, 374(2065), p. 20150202. Available at: https://doi.org/10.1098/rsta.2015.0202.

Riskin, D.K. *et al.* (2008) ‘Quantifying the complexity of bat wing kinematics’, *Journal of Theoretical Biology*, 254(3), pp. 604–615. Available at: https://doi.org/10.1016/j.jtbi.2008.06.011.

Stowers, A.K., Matloff, L.Y. and Lentink, D. (2017) ‘How pigeons couple three-dimensional elbow and wrist motion to morph their wings’, *Journal of The Royal Society Interface*, 14(133), p. 20170224. Available at: https://doi.org/10.1098/rsif.2017.0224.
